# Severity of parkinsonism associated with environmental manganese exposure

**DOI:** 10.1186/s12940-021-00712-3

**Published:** 2021-03-15

**Authors:** Brad A. Racette, Gill Nelson, Wendy W. Dlamini, Pradeep Prathibha, Jay R. Turner, Mwiza Ushe, Harvey Checkoway, Lianne Sheppard, Susan Searles Nielsen

**Affiliations:** 1 Department of Neurology, Washington University School of Medicine, 660 South Euclid Avenue, Campus Box 8111, 63110 St. Louis, Missouri, USA; 2 School of Public Health, Faculty of Health Sciences, University of the Witwatersrand, 7 York Road, 2193 Parktown, South Africa; 3 Research Department of Infection & Population Health, UCL Institute for Global Health, University College London, London, UK; 4 Department of Energy, Environmental, and Chemical Engineering, Washington University, Campus Box 1180, One Brookings Drive, 63130 St. Louis, Missouri, USA; 5 Department of Family Medicine & Public Health, University of California, 9500 Gilman Drive, # 0725, La Jolla, 92093-0725 San Diego, California USA; 6 Departments of Biostatistics and Environmental and Occupational Health Sciences, University of Washington, Box 357232, Washington, 98195 Seattle, USA

**Keywords:** Case control studies, Parkinson disease, Parkinsonism, Manganese

## Abstract

**Background:**

Exposure to occupational manganese (Mn) is associated with neurotoxic brain injury, manifesting primarily as parkinsonism. The association between environmental Mn exposure and parkinsonism is unclear. To characterize the association between environmental Mn exposure and parkinsonism, we performed population-based sampling of residents older than 40 in Meyerton, South Africa (N = 621) in residential settlements adjacent to a large Mn smelter and in a comparable non-exposed settlement in Ethembalethu, South Africa (N = 95) in 2016–2020.

**Methods:**

A movement disorders specialist examined all participants using the Unified Parkinson Disease Rating Scale motor subsection part 3 (UPDRS3). Participants also completed an accelerometry-based kinematic test and a grooved pegboard test. We compared performance on the UPDRS3, grooved pegboard, and the accelerometry-based kinematic test between the settlements using linear regression, adjusting for covariates. We also measured airborne PM_2.5_-Mn in the study settlements.

**Results:**

Mean PM_2.5_-Mn concentration at a long-term fixed site in Meyerton was 203 ng/m^3^ in 2016–2017 – approximately double that measured at two other neighborhoods in Meyerton. The mean Mn concentration in Ethembalethu was ~ 20 times lower than that of the long-term Meyerton site. UPDRS3 scores were 6.6 (CI 5.2, 7.9) points higher in Meyerton than Ethembalethu residents. Mean angular velocity for finger-tapping on the accelerometry-based kinematic test was slower in Meyerton than Ethembalethu residents [dominant hand 74.9 (CI 48.7, 101.2) and non-dominant hand 82.6 (CI 55.2, 110.1) degrees/second slower]. Similarly, Meyerton residents took longer to complete the grooved pegboard, especially for the non-dominant hand (6.9, CI -2.6, 16.3 s longer).

**Conclusions:**

Environmental airborne Mn exposures at levels substantially lower than current occupational exposure thresholds in the United States may be associated with clinical parkinsonism.

## Background

Manganese (Mn) is an essential trace element [1] but also a neurotoxin at higher levels. Routes of entry are oral, respiratory, and, possibly, trans-olfactory. Mn that bypasses the liver is actively transported across the blood-brain barrier and appears to accumulate in the basal ganglia [2]. Oral Mn uptake is tightly regulated to maintain homeostatic Mn blood levels, so neurotoxicity in adults appears to be related primarily to inhaled Mn [3]. Millions of people worldwide are exposed to airborne environmental Mn due to fossil fuel combustion, air erosion of Mn-laden soils proximate to mining operations, and industrial stack emissions from high temperature industrial processes, such as smelting and steelmaking. Numerous studies demonstrate an association between occupational Mn exposure and motor dysfunction [4–8]. There is also evidence of Mn-related motor dysfunction [9–17], in relation to environmental Mn exposure in adults, but fewer studies have found clinically relevant motor health effects [12, 18]. We have previously shown that Mn-exposed workers have Mn-dose-dependent parkinsonism [7] and dopaminergic dysfunction [19–21], at estimated mean airborne Mn concentrations ranging from 0.0175 to 0.14 mg/m^3^ over the course of a work shift. This and other studies [8, 22] suggest that there are adverse neurologic health effects from Mn exposures below the American Conference of Governmental Industrial Hygienists (ACGIH) threshold limit value for Mn of 0.1 mg/m^3^ [23]. The current United States (U.S.) Environmental Protection Agency (EPA) lowest observed adverse effect level (LOAEL) of Mn is 0.05 mg/m^3^, and was derived from findings from an occupational study [24]. We sought to examine whether we could detect motor health effects from ambient industrial Mn exposure in South Africa. We hypothesized that individuals with relatively high Mn environmental inhalational exposures would have poorer scores on clinically relevant measures of parkinsonism than those with lower exposures.

## Methods

### Participants

All participants lived in one of two communities in Gauteng province, South Africa at the time of enrollment, between 2016 and 2020.Participants in the Mn-exposed community, Meyerton, lived in one of three settlements (Old Sicelo, New Sicelo, or Noldick). This community is located in the Midvaal municipality, within 5 km of one of the world’s largest Mn smelters, which has been in operation since 1951. Participants from the reference community lived in Ethembalethu, a settlement located approximately 70 km northwest of Meyerton, in the Mogale City municipality, with no nearby Mn smelting or mining operations. We chose Ethembalethu as the reference settlement due to its location in a non-industrial area, outside of Johannesburg, but otherwise largely similar sociodemographics (Table 1). Most notably, the selected Meyerton-based and Ethembalethu settlements are government-subsidized housing communities, so residents must meet the same income criteria to be allowed to live in these settlements.

**Table 1:** Characteristics of residents and households, by municipality^a^

	Midvaal (includes Meyerton settlements Noldick, Old Sicelo, New Sicelo)	Mogale City (includes Ethembalethu)
Households		
Formal dwelling, %	80.2	73.5
Female-headed, %	26.3	31.2
Owned,^b^ %	42.6	39.0
Utilities, %		
Piped water inside dwelling	64.9	54.8
Flush toilet/sewer	58.0	78.2
Electricity for lighting	79.3	85.9
Weekly removal of refuse	82.1	79.7
Residents		
Total population, N	95,301	362,422
Population density, persons/km^2^	55	270
Sex, %		
Female	48.4	49.0
Male	51.6	51.0
Race, %		
Black	58.4	75.6
White	38.7	21.0
Other	2.9	3.5
Age, years, %		
0–14	23.2	23.7
15–64	70.5	71.7
≥65	6.3	4.6
Dependency ratio^c^	41.9	39.4
Education (age ≥ 20 years), %		
No schooling	5.2	4.7
Primary/secondary	47.5	48.6
Matric (high school)	32.1	32.6
Higher education	15.2	14.1
Unemployed, %		
Overall	18.8	24.6
Youth	25.4	32.3
No income, %	14.5	15.5

^a^ Statistics South Africa, 2011 ()

^b^ Owned includes “paying off” a loan for purchase of the home

^c^ All ratios presented are multiplied by 100. The dependency ratio is the number of residents’ age 0–14 or ≥ 65, divided by residents’ age 15–64

Our research personnel recruited participants by visiting a preselected, population-based sample of homes in each settlement to attempt to recruit adults who met all inclusion criteria, as detailed below. For two of the three Meyerton-based settlements (New Sicelo and Noldick), we preselected every other residence using a municipality map. Research personnel attempted to recruit eligible adults in each residence to participate in the study. If no one was home, or if there were no eligible adults in the residence, the research personnel attempted to recruit the residence to the left of the preselected home. If no one was home or eligible in that residence, they proceeded to the next preselected home on the map. Because there were fewer residences in Old Sicelo than in the other two areas, research personnel attempted to recruit participants from every residence in that settlement. The reference community, Ethembalethu, was smaller than the Meyerton-based settlements, so we attempted to recruit every adult resident who met the study criteria, using the same door-to-door approach.

Inclusion criteria included current residence in the selected Meyerton-based settlements or Ethembalethu, age ≥ 40, and ability to provide informed consent. After initial recruitment based upon these inclusion criteria, and completion of grooved pegboard (GP) testing, accelerometry-based kinematic testing, and the study questionnaire in the home, participants were asked to come for a second visit (“phase 2”) to a local community center to be examined by a neurologist at a later time.After this clinical assessment phase 2 visit, enrolled participants were then excluded if they had neurologic co-morbidities that made testing unreliable or were using a dopamine receptor blocking medication. Otherwise, we did not select participants with regard to any health outcomes or occupational exposures, and generally, participants only had non-occupational exposure to Mn.

### Assessment of UPDRS3 score and subscores

One movement disorder specialist (B.A.R.) examined all participants for Parkinson disease (PD) and, more generally, signs of parkinsonism, using the Unified Parkinson Disease Rating Scale motor subsection part 3 (UPDRS3) [25]. The complete examination occurred while blinded to results on the GP test, accelerometry-based kinematic testing, and cumulative Mn exposure, i.e., current residence location within the respective community, past residential histories, and occupational histories. In addition to the UPDRS3 total score, we combined selected UPDRS3 subscores [7], as secondary outcomes, to determine if environmental Mn exposure was associated with specific clinical signs. In order to ensure that participants could be included even when a limb was severely injured or missing, or had a medical condition that precluded the pull test to assess balance, we imputed one or more missing subscores, when possible, using other subscores as predictors in linear regression models based on all participants with complete UPDRS3 subscores.

### Grooved pegboard and kinematic testing

Participants completed selected motor tasks, as additional secondary outcomes, in their home at the time of recruitment. These included two tasks that assess fine motor function: the GP test and an accelerometry-based kinematic test (hereafter “kinematic test”) that replicated the finger-tapping task from the UPDRS3 exam. For GP testing, we used a standard GP device (Lafayette Instrument Company, Lafayette, Indiana) and followed published testing procedures [26]. We recorded the time to place the 25 pegs for each hand up to 300 s. Trained research personnel administered the kinematic test by placing a wireless motion sensor (Kinesia™, Great Lakes NeuroTechnologies, Independence, Ohio) [27–31] on the top of the participant’s index finger. The Kinesia Motion Sensory device is comprised of a triaxial accelerometer and triaxial gyroscope, allowing the measurement of acceleration (linear) and velocity (angular), respectively, along all three axes (x, y, and z) at 64 Hz. We recorded the digitized signals on a computer tablet, installed with motion capture software (Great Lakes NeuroTechnologies, Independence, Ohio). Participants were asked to complete three 12-second trials for each hand for a finger-tapping task.Each participant tapped his/her index finger and thumb together, while keeping the other fingers stable and the elbow extended.Participants were instructed to perform the finger-tapping task with as large an amplitude and as fast as possible. We then processed the kinematic data using code we developed in Stata version MP 14.2 (StataCorp, College Station, Texas) [32] and validated this Stata processed data against manually processed data (Spearman’s ρ > 0.99). We used the mean angular velocity in degrees/second, hereafter referred to as mean velocity, across all three trials for the respective hand (dominant or non-dominant). We used self-reported handedness to classify the motor tasks as dominant or non-dominant. Even after age-adjustment, both motor tasks in each hand were strongly associated with the UPDRS3 (all *P* values < 0.001).

### Assessment of mn exposure

We used community (Meyerton, Ethembalethu) as an indicator of Mn exposure status. To verify and quantify potential differences in airborne Mn exposure, we measured ambient Mn concentrations in both communities. We collected fine particulate matter (PM_2.5_, particles with aerodynamic diameter ≤ 2.5 μm) on Teflon® filters (Measurement Technology Corporation, Minneapolis, MN) using air samplers with PM_2.5_ inlets (Model PQ100, Mesa Labs, Butler, NJ) operating continuously for two- to three-days for each sample. Long-term routine air sampling at a fixed site in the Meyerton settlement of Noldick began in October 2015 and was completed in May 2018. For the two-year period 2016–2017, 47 % of all hours were represented (*n* = 158 filters). We assessed spatial variability across the Meyerton-based settlements by collecting samples concurrently in Old Sicelo and Noldick (October 2018-February 2019, *n* = 37 filters), and New Sicelo and Noldick (September 2017-May 2018 and October 2018, n = 55 filters). We conducted air sampling in Ethembalethu in January-October 2020 (*n* = 68 filters) with no concurrent sampling in Meyerton. Filter membranes were digested using a MARS 6™ microwave digestion system (CEM, Matthews, NC) using a validated protocol [33]. We filtered these digestates through 0.45 μm (pore size) nylon syringe filters (VWR, Radnor, PA) and diluted them with deionized water (≥ 18.2 MΩ/cm resistivity, MilliQ Water Purification System, EMD Millipore, Burlington, MA). Mn was quantified using an inductively coupled plasma-mass spectrometer (NexION® 2000, Perkin-Elmer, Norwalk, CT). The limit of detection for Mn was 0.056 ng/m^3^ in PM_2.5_ [34]. Instrument performance was validated using NIST 1648a Urban Particulate Matter (Sigma-Aldrich, St. Louis, MO), yielding Mn recovery of 96.9 ± 8.4 %.

### Statistical analysis

We performed all statistical analyses using Stata version MP 14.2 [32]. We used linear regression with each of the motor outcomes as continuous dependent variables. Mn exposure, as assessed by whether the residence was in the exposed (Meyerton) or non-exposed (Ethembalethu) community, was the independent variable of primary interest. Our primary motor outcome was the total UPDRS3 score. Given the known strong, positive association between age and both UPDRS3 scores [7] and other motor outcomes, we adjusted *a priori* for age in all models. We retained age as a continuous variable and adjusted for age using natural cubic splines with five knots, following Harrell’s placement method, i.e., knots equally spaced at the 5th, 27.5th, 50th, 72.5th and 95th percentiles [35]. In practice, five knots are considered a good choice to model the overall shape of a parameter for sample sizes ≥ 100 [35, 36]. We also examined the effects of adjustment for sex, cigarette smoking, and alcohol use, with the latter two as trichotomous variables (never, former, current use). These demographic variables are associated with PD [37–39] and therefore might also be associated with UPDRS3 scores and other motor outcomes. We conducted three additional sensitivity analyses. First, we excluded participants with imputed UPDRS3 subscores to assess the stability of the results.Second, because kinematic test data were not available for some participants, we repeated the kinematic analysis while applying inverse probability weighting to give greater weight to participants with characteristics associated with missing kinematic data (as estimated by a logistic regression model that predicted missingness of kinematic data).Finally, we excluded participants with any current or previous occupational Mn exposure.In addition, through exploratory analyses we investigated whether restriction of Meyerton participants to those who had lived in the same home in Meyerton since before 2008 (when Mn production at the smelter decreased due to a recession) revealed stronger associations for the motor outcomes. For all analyses, we considered a two-sided P value of 0.05 as statistically significant, evidenced by the exclusion of zero from the 95 % CI for the β coefficient, i.e., adjusted mean difference between Meyerton and Ethembalethu.

## Results

**Fig. 1:**
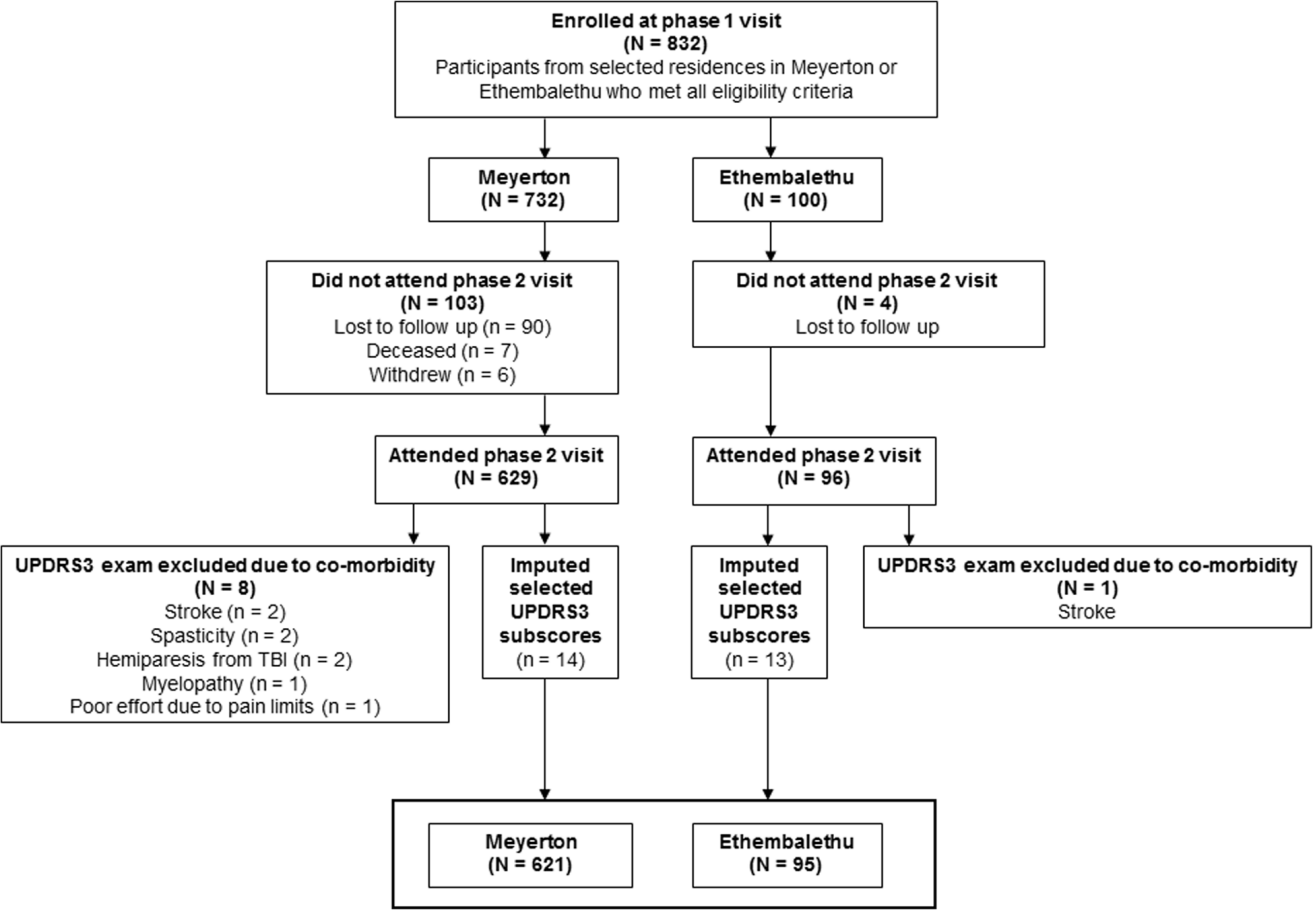
Participating residents (*N* = 716) of Meyerton and Ethembalethu, Gauteng province, South Africa, 2016–2020. Eligible participants were aged ≥ 40, and able to provide informed consent. Eligible participants with neurologic co-morbidities, and those without a phase 2 clinical assessment and consequently did not have a UPDRS3 exam, were excluded.Abbreviations: UPDRS3 = Unified Parkinson Disease Rating Scale motor subsection part 3.

Out of the 666 homes we visited in Meyerton, 462 (69.4 %) had at least one eligible adult who agreed to participate; and out of the 108 homes we visited in Ethembalethu, 79 (73.1 %) had at least one eligible adult who agreed to participate.Initially, we recruited 832 eligible participants (732 in Meyerton, 100 in Ethembalethu) (Fig. 1). The median time between the first and second visits in Meyerton and Ethembalethu was 49 and 3 days, respectively. Of those who were enrolled at the first visit, 629 (85.9 %) and 96 (96.0 %), respectively, attended the phase 2 clinical assessment visit in Meyerton and Ethembalethu. After excluding some participants for co-morbidities, we retained 621 (98.7 %) and 95 (99.0 %) eligible participants in each of the communities, respectively, who had complete UPDRS3 scores, following imputation of selected subscores for 17 individuals.We obtained GP testing data from both hands for 605 (97.4 %) and 93 (97.9 %) participants, respectively, and we obtained kinematic test data from both hands for 346 (55.7 %) Meyerton participants and 91 (95.8 %) Ethembalethu participants.Most participants in both communities were Black (98.9 % in Meyerton and 97.9 % in Ethembalethu). Other demographic characteristics of the participants and their communities are in Tables 1 and 2, respectively.

**Table 2:** Characteristics of participants, overall and by community, Gauteng province, South Africa, 2016–2020

	All participants *N* = 716	Mn smelter community (Meyerton) *N* = 621	Reference community (Ethembalethu) *N* = 95
	**n (%)**	**n (%)**	**n (%)**
Sex			
Female	405 (56.6)	339 (54.6)	66 (69.5)
Male	311 (43.4)	282 (45.4)	29 (30.5)
Race^a^			
Black	706 (98.7)	614 (98.9)	92 (97.9)
Other	9 (1.3)	7 (1.1)	2 (2.1)
Language^b^			
Sesotho	366 (51.3)	352 (57.0)	14 (14.7)
IsiXhosa	107 (15.0)	99 (16.0)	8 (8.4)
IsiZulu	107 (15.0)	91 (14.7)	16 (16.8)
Setswana	44 (6.2)	14 (2.3)	30 (31.6)
Sepedi	25 (3.5)	19 (3.1)	6 (6.3)
Other	64 (9.0)	43 (7.0)	21 (22.1)
Education^c^			
None/non-formal schooling	100 (14.6)	92 (15.5)	8 (8.9)
Primary	252 (36.8)	214 (36.0)	38 (42.2)
Secondary	226 (33.0)	198 (33.3)	28 (31.1)
Matric or higher	107 (15.6)	91 (15.3)	16 (17.8)
Unemployed^d^	354 (50.6)	317 (52.3)	37 (39.4)
Smoking cigarettes^e^			
Never	493 (69.3)	413 (66.6)	80 (87.9)
Former	57 (8.0)	56 (9.0)	1 (1.1)
Current	161 (22.6)	151 (24.4)	10 (11.0)
Alcohol use			
Never	367 (51.3)	301 (48.5)	66 (69.5)
Former	113 (15.8)	101 (16.3)	12 (12.6)
Current	236 (33.0)	219 (35.3)	17 (17.9)
Ever Mn occupational exposure	14 (2.0)	14 (2.3)	0 (0.0)
Current Mn occupational exposure	2 (0.3)	2 (0.3)	0 (0.0)
	**Mean (SD)**	**Mean (SD)**	**Mean (SD)**
Age, years	51.8 (9.2)	51.3 (9.2)	55.3 (8.7)
Minimum	40	40	40
Median	50	49	55
Maximum	97	97	84

Abbreviations: *Mn* manganese

^a^ Percent excludes 1 participant from Ethembalethu with missing data. Other is White or of mixed race.

^b^ Percent excludes 3 participants from Meyerton with missing data. Other languages are Xitsonga, Afrikaans, SiSwati, Tshivenda, and English.

^c^ Percent excludes 31 participants with missing data (5 from Ethembalethu, and 26 from Meyerton); where primary is grades 1 – 7, secondary is grades 8 – 11, and matric is grade 12.

^d^ Percent excludes 16 participants with missing data (1 from Ethembalethu, and 15 from Meyerton).

^e^ Percent excludes 5 participants with missing data (4 from Ethembalethu, and 1 from Meyerton).

In Meyerton, the two-year (2016–2017) mean PM_2.5_-Mn concentration from the long-term particulate matter air sampling in Noldick was 203 ng/m^3^. Based on the concurrent sampling in Noldick and the other two settlements in Meyerton, the mean was approximately twice that for both Old Sicelo and New Sicelo; the PM_2.5_-Mn ratio of means were 0.45 at Old Sicelo and 0.65 at New Sicelo, compared to Noldick. The PM_2.5_-Mn mean concentration in Ethembalethu (year 2020) was 10 ng/m^3^, i.e., ~ 20 times lower than the concentrations in Noldick.

**Fig. 2:**
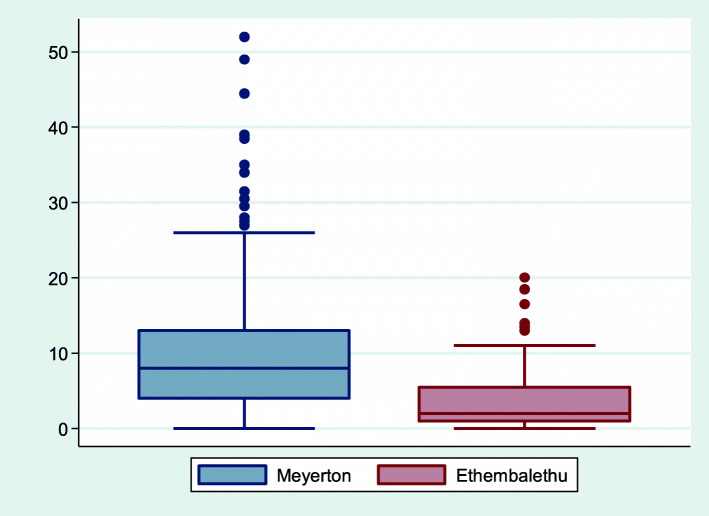
**Title**: Box and whisker plot of UPDRS3 scores by community, Gauteng province, South Africa, 2016–2020. Figure demonstrates median, interquartile range, and overall UPDRS3 score range, including outliers, and shows greater severity of parkinsonism in the Mn smelter community of Meyerton (*N* = 621) as compared to the reference community of Ethembalethu (*N* = 95), Gauteng province, South Africa, 2016-2020. Abbreviations: Mn = manganese; UPDRS3 = Unified Parkinson Disease Rating Scale motor subsection part 3.

The mean UPDRS3 score was higher in Meyerton than Ethembalethu residents (Fig. 2), with mean UPDRS3 scores of 9.3 (SD 7.2) in Meyerton and 3.7 (SD 4.1) in Ethembalethu (Table 3). After accounting for the slightly younger mean age of residents from Meyerton compared to Ethembalethu, residents of Meyerton had a UPDRS3 score 6.6 (CI 5.2, 7.9) points higher than residents of Ethembalethu, on average (Table 4). This difference was driven by higher subscores for upper limb bradykinesia and rigidity and lower limb bradykinesia and rigidity, with each individual subscore in these categories contributing approximately 0.5 points, on average, for Meyerton vs. Ethembalethu residents (Table 4). The association between community and total UPDRS3 score was not changed materially with adjustment for factors in addition to age, with only 4.5 % attenuation after adjustment for sex, smoking, and alcohol. Results were consistent when excluding 17 participants with missing UPDRS3 subscores and, separately, 14 participants with any history of occupational Mn exposure. The association between community and UPDRS3 did not differ according to age, sex, smoking, or alcohol use (all interaction P values > 0.05). In addition to the differences in UPDRS3 score by community, Meyerton residents had a slower mean velocity on the kinematic test than Ethembalethu residents [74.9 (CI 48.7, 101.2) degrees/second slower for the dominant hand, and 82.6 (CI 55.2, 110.1) degrees/second slower for the non-dominant hand] (Tables 4 and 5). This association was attenuated somewhat, but clearly remained, when we applied inverse probability weighting to address missingness of kinematic test data. Specifically, Meyerton residents were 58.7 (CI 27.6, 89.7) and 65.7 (CI 32.4, 99.1) degrees/second slower in the dominant and non-dominant hand, respectively, compared to Ethembalethu residents. Similarly, there was a suggestion that Meyerton residents took longer to complete the GP test, for the non-dominant hand (6.9, CI -2.6, 16.3 s longer). As with the UPDRS3 scores, results for the kinematic test and GP test were not materially changed by adjusting for additional covariates beyond age. Associations between community and each of the motor outcomes were not markedly different when we restricted the Meyerton residents to those who had lived in their homes since before 2008.

**Table 3:** UPDRS3 motor outcomes, overall and by community, Gauteng province, South Africa, 2016–2020

	All participants *N* = 716	Mn smelter community (Meyerton) *N* = 621	Reference community (Ethembalethu) *N* = 95
	**n (%)**	**n (%)**	**n (%)**
Total UPDRS3 score ≥ 15^a^	123 (17.2 %)	120 (19.3 %)	3 (3.2 %)
	**Mean (SD)**	**Mean (SD)**	**Mean (SD)**
UPDRS3^a^			
Total score	8.5 (7.1)	9.3 (7.2)	3.7 (4.1)
Minimum	0	0	0
Median	7	8	2
Maximum	52	52	20
Upper limb bradykinesia^b^	3.7 (3.2)	4.0 (3.2)	1.5 (2.1)
Minimum	0	0	0
Median	3.5	4	1
Maximum	17	17	8
Upper limb rigidity^c^	1.0 (1.3)	1.1 (1.3)	0.4 (0.7)
Minimum	0	0	0
Median	0	0	0
Maximum	5	5	3
Lower limb bradykinesia^c^	0.7 (1.1)	0.8 (1.2)	0.3 (0.6)
Minimum	0	0	0
Median	0	0	0
Maximum	6	6	2
Lower limb rigidity^c^	1.0 (1.4)	1.1 (1.4)	0.3 (0.7)
Minimum	0	0	0
Median	0	0	0
Maximum	6	6.0	2.5
Rest tremor^d^	0.04 (0.3)	0.1 (0.3)	0.0 (0.0)
Minimum	0	0	0
Median	0	0	0
Maximum	3	3	0
Action tremor^c^	0.1 (0.5)	0.2 (0.5)	0.04 (0.2)
Minimum	0	0	0
Median	0	0	0
Maximum	4	4	2
Axial signs^e^	1.9 (2.4)	2.0 (2.4)	1.2 (1.9)
Minimum	0	0	0
Median	1	2	1
Maximum	24	24	13

Abbreviations: *Mn* manganese; *UPDRS3* Unified Parkinson Disease Rating Scale motor subsection part 3

^a^ Poorer motor performance is indicated by greater UPDRS3 scores.

^b^ Sum of six UPDRS3 subscores: Finger-tapping, hand rotations, and rapid arm movements for each limb.

^c^ Sum of the two UPDRS3 subscores (one for each limb).

^d^ Sum of five UPDDRS3 subscores: Upper limbs, lower limbs, and face.

^e^ Sum of eight UPDRS3 subscores: Speech, facial expression, neck rigidity, difficulty arising from a chair, posture, gait, postural instability, and global bradykinesia.

**Table 4:** Difference in motor outcomes associated with Mn exposure by community, Gauteng province, South Africa, 2016–2020

	Mean difference between Mn smelter community vs. reference community in specified motor outcome (95 % CI)^a^		
	**Unadjusted**	**Age-adjusted**	**Fully-adjusted**
UPDRS3, total score^b^	5.6 (4.1, 7.1)	6.6 (5.2, 7.9)	6.3 (4.9, 7.7)
UPDRS3 subscores^b^			
Upper limb bradykinesia^c^	2.5 (1.8, 3.1)	2.9 (2.2, 3.5)	2.9 (2.2, 3.6)
Upper limb rigidity^d^	0.8 (0.5, 1.0)	0.8 (0.6, 1.1)	0.8 (0.5, 1.1)
Lower limb bradykinesia^d^	0.5 (0.2, 0.7)	0.6 (0.4, 0.8)	0.6 (0.3, 0.8)
Lower limb rigidity^d^	0.8 (0.6, 1.1)	0.9 (0.6, 1.2)	0.9 (0.6, 1.2)
Rest tremor^e^	0.1 (-0.01, 0.1)	0.1 (-0.005, 0.1)	0.04 (-0.02, 0.1)
Action tremor^d^	0.1 (0.01, 0.2)	0.1 (0.01, 0.2)	0.1 (-0.02, 0.2)
Axial signs^f^	0.8 (0.3, 1.3)	1.1 (0.7, 1.6)	1.0 (0.5, 1.5)
Grooved pegboard, time (seconds)^b^			
Dominant hand	-7.6 (-17.0, 1.9)	1.6 (-7.2, 10.5)	0.9 (-8.2, 10.0)
Non-dominant hand	-4.0 (-14.3, 6.3)	6.9 (-2.6, 16.3)	7.5 (-2.2, 17.1)
Kinematic testing, finger-tapping mean velocity (degrees/second)^b^			
Dominant hand	-68.4 (-93.9, -43.0)	-74.9 (-101.2, -48.7)	-69.0 (-96.8, -41.3)
Non-dominant hand	-72.8 (-99.6, -46.1)	-82.6 (-110.1, -55.2)	-70.6 (-99.6, -41.6)

Abbreviations: *Mn* manganese; *UPDRS3* Unified Parkinson Disease Rating Scale motor subsection part 3

^a^ Based on 716 participants (621 in Meyerton and 95 in Ethembalethu). Age adjustment was using age as a continuous variable with natural cubic splines with five knots (5th, 27.5th, 50th, 72.5th, and 95th percentiles) as per Harrell’s placement method [35]. Fully adjusted means adjusted for age, sex, cigarette smoking (ever, former, current), and alcohol use (ever, former, current) (with all results confirmed in models with smoking as a dichotomous variable due to the small number of former smokers)

^b^ Poorer motor performance is indicated by greater UPDRS3 scores, greater grooved pegboard times, and lower finger-tapping velocities

^c^ Sum of six UPDRS3 subscores: Finger-tapping, hand rotations, and rapid arm movements for each limb

^d^ Sum of the two UPDRS3 subscores (one for each limb).

^e^ Sum of five UPDRS3 subscores: Upper limbs, lower limbs, and face

^f^ Sum of eight UPDRS3 subscores: Speech, facial expression, neck rigidity, difficulty arising from a chair, posture, gait, postural instability, and global bradykinesia

**Table 5:** Grooved pegboard and kinematic motor outcomes, overall and by community, Gauteng province, South Africa, 2016–2020

	All participants *N* = 716	Mn smelter community (Meyerton) *N* = 621	Reference community (Ethembalethu) *N* = 95
	**Mean (SD)**	**Mean (SD)**	**Mean (SD)**
Grooved pegboard test, time (seconds)^a^			
Dominant hand	108.0 (43.1)	107.0 (43.0)^b^	114.6 (43.4)^c^
Minimum	43.2	43.2	59.2
25th percentile	80.2	79.9	82.0
Median	93.8	93.5	114.0
75th percentile	124.8	120.1	141.2
Maximum	300.0	300.0	300.0
Non-dominant hand	118.0 (47.1)	117.5 (47.4)^b^	121.4 (44.8)^c^
Minimum	51.4	51.4	60.0
25th percentile	86.9	87.1	85.1
Median	103.8	103.2	114.7
75th percentile	131.7	130.3	150.3
Maximum	300.0	300.0	241.1
Kinematic test – finger-tapping mean velocity (degrees/second)^a^			
Dominant hand	312.4 (113.2)	298.2 (106.0)^b^	366.6 (123.5)^c^
Minimum	59.0	59.0	121.9
25th percentile	222.7	217.3	275.9
Median	302.7	289.7	355.8
75th percentile	384.6	362.2	445.5
Maximum	679.0	590.5	679.0
Non-dominant hand	353.3 (119.2)	338.2 (111.7)^b^	411.0 (129.5)^c^
Minimum	87.8	87.8	168.5
25th percentile	271.2	262.6	316.3
Median	342.7	326.8	412.9
75th percentile	426.3	399.4	509.4
Maximum	764.4	727.0	764.4

Abbreviations: *Mn* manganese

^a^ Poorer motor performance is indicated by greater grooved pegboard times and lower finger-tapping velocities

^b^ Excludes 16 participants (grooved pegboard in dominant hand and non-dominant hand) and 275 participants (kinematic test in dominant and non-dominant hand) with missing data

^c^ Excludes two participants (grooved pegboard in dominant and non-dominant hand) and four participants (kinematic test in dominant and non-dominant hand) with missing data

Five (0.8 %) participants from Meyerton and no participants from Ethembalethu had PD (Fisher’s exact two-sided *P* value = 1.00, Fisher’s exact one-sided *P* value = 0.49).

## Discussion

This study provides evidence of an association between environmental Mn exposure and parkinsonian motor dysfunction. We chose a clinically relevant primary motor assessment of parkinsonism, the UPDRS3, which we previously demonstrated to be associated with PD-specific quality of life in language-adapted questionnaires in this population [40]. Interestingly, the mean UPDRS3 in the Meyerton community was similar to that reported in several occupationally exposed Mn populations [7, 8, 41]. This is notable because these contemporaneous worker populations experience estimated 8-hour time-weighted mean Mn exposures of 0.0175-0.23 ± 0.18 mg/m^3^ [7, 8, 41], whereas ambient Mn concentration levels in our environmentally exposed population appeared to be substantially lower (0.00075–0.0026 mg/m^3^), at least during the study period. The longer time that Meyerton vs. Ethembalethu residents took to complete the GP test, and the slower finger-tapping on the accelerometry-based kinematic test, provide objective confirmation of the primary UPDRS3 results. Strengths of our study include a rigorous population-based sampling approach in the two similar communities and the use of standardized and clinically relevant motor outcomes. Our large study, with expert neurological assessments and measurement of airborne Mn, provides evidence of an association between parkinsonism and environmental Mn exposure.

The neurologic health effects we observed in our Mn-exposed community during the monitoring period occurred in the setting of two-year (2016–2017) average ambient PM_2.5_-Mn up to 215 ng/m^3^, with evidence that ambient concentrations were approximately half this value in the other two settlements from which we recruited participants in the exposed community. These ambient levels of Mn contrast with the U.S. EPA LOAEL of 0.05 mg/m^3^ (50,000 ng/m^3^) and indicate there may be neurologic health effects associated with exposures substantially lower than the LOAEL for PM_2.5_-Mn. One important caveat is that we measured PM_2.5_-Mn in Meyerton from 2015 to 2019 and Mn production at the smelter then was lower than it was before the 2008 recession. As a result, we may be underestimating the air concentrations to which our Meyerton participants were exposed in earlier years. Nevertheless, even Mn exposure an order of magnitude greater than our measured PM_2.5_-Mn levels still represents an exposure level far below that measured in the occupational study on which the current LOAEL for PM_2.5_-Mn is based [24].

Our study findings are consistent with several epidemiologic studies, using various methods, which demonstrate motor dysfunction in relation to environmental Mn exposure [16]. The study most similar to ours methodologically observed modest but significant differences in UPDRS3 in residents of Marietta, OH, relative to a reference community, of 0.22 points [42]. While other prior studies used different methods of either assessing Mn or motor outcomes, our study adds to the literature by using a clinically valid and relevant quantitative measure of parkinsonism [25, 41]. One study in Canada demonstrated an association between computer-based tests of tremor, pointing, and pronation/supination hand movements and blood Mn [10, 11]. In a follow-up study of the Mn-exposed Ohio communities (Marietta and East Liverpool), investigators reported an association between Mn exposure and a computer-based tremor and finger-tapping performance (inverse) [14, 15]. Modeled PM_2.5_-Mn exposures ranged from 1 to 340 ng/m^3^. An Italian study of parkinsonism, defined by use of levodopa, found that municipalities with historic industrial Mn exposures and with higher soil concentrations of Mn had higher standardized morbidity ratios for parkinsonism than other regions of Italy [12]. This same group investigated motor function, using the motor coordination tests in the Luria Nebraska Neuropsychological Battery, in residents from the same region of Italy, and found an association with PM_10_-Mn, though the mean PM_10_-Mn was similar between the Mn-exposed and reference regions [13]. While these environmental studies provide consistent evidence of motor dysfunction in relation to even lower level Mn exposures than found in occupational settings, the pathophysiology of this motor dysfunction is largely unknown. Studies in occupational cohorts demonstrate evidence of a dose-dependent association between occupational Mn-dose exposure and dopaminergic dysfunction [19, 20, 43] and thalamic gamma aminobutyric acid (GABA) levels [8, 44]. The mechanism of this dysfunction may be due to Mn-induced neuroinflammation [45, 46]. Whether these same mechanisms apply to the much lower environmental exposures will require further study.

We focused on parkinsonism instead of PD, given the relatively low prevalence of PD.With that said, we did identify five participants with PD from our randomly sampled Meyerton population and no cases in Ethembalethu, raising the possibility that PD may be relatively common in this community with high levels of inhalational Mn exposure relative to our reference region, in South Africa. However, our study was not powered to test that association, and any difference in PD prevalence might represent a chance finding. Nonetheless, our results are consistent with a previous study in which we used geographic information systems to investigate the geography of incident PD in the U.S. [18]. In that study, we used Medicare claims data to identify PD cases and calculated county-level PD incidence. We observed a higher incidence of neurologist-diagnosed PD in urban U.S. counties with high Mn release, compared with urban counties with none, which was specific to Mn emissions [18]. Using the 2005 EPA National-Scale Air Toxics Assessment (NATA), we estimated that the contemporary Mn exposures in these regions corresponded to approximately 0.00005–0.0008 mg/m^3^, levels substantially below the EPA LOAEL. The results of our previous geographic study are largely consistent with the estimates of environmental Mn exposure at which health effects may be seen, based upon the findings in this current study.

Our research participants reside in an environment of poverty and social neglect. The adult residents of these communities endure high rates of unemployment and many had very little education due to Apartheid era policies. The location of the Meyerton settlement near a source of industrial pollution is similar to the placement of low-cost housing in the U.S. and throughout the world. One unique aspect of our study is our success in recruiting a population of Black African residents in impoverished communities to investigate an environmental parkinsonism hypothesis. While we encountered many challenges, some unique to South Africa, when implementing this protocol, we were able to recruit successfully what is possibly the largest Black African parkinsonism cohort ever established. We anticipate future studies will continue to build on this success.

As with any study, there are some limitations. First, we only present mean community exposures. Ongoing efforts to model individual level inhalational Mn exposures from the smelter and other sources of airborne Mn should provide further insight into dose-response relations. Second, blinding the UPDRS3 to community of residence (Meyerton vs. Ethembalethu), i.e., Mn exposure status, was not possible, so we included additional motor assessments as an objective means to attempt to confirm the UPDRS3 results. These assessments, the accelerometry-based kinematic and GP tests, confirmed poorer motor performance among residents from Meyerton vs. Ethembalethu. Third, while overall recruitment was quite successful, not everyone invited to participate was willing to participate in the study, so there could be some bias toward those with or without symptoms participating in our study.However, we are not aware of incentives or disincentives for symptomatic or asymptomatic residents of Meyerton to participate and/or symptomatic or asymptomatic residents of Ethembalethu to not participate. Although we found that residents of both communities were overwhelmingly supportive of the research, we did not include anyone in the study who was not selected through the population-based sampling method. Fourth, this study focused on a specific Mn-exposed community; we do not know if these results are generalizable to other similar communities with environmental exposure to Mn, even though the exposure levels overlap with those in some U.S. populations. Finally, we acknowledge that inter-individual variation in terms of actual Mn exposures and other factors that might affect Mn dose within the brain could be relevant to the motor outcomes of interest in this study.

We observed a strong relation between residential exposure to environmental Mn and parkinsonian motor signs at air concentrations substantially lower than international occupational thresholds. Although further detailed exposure quantification is ongoing, these results suggest that current U.S. and international Mn exposure limits may need to be revised.

## Conclusions

In this large epidemiological study of environmental Mn exposure in South Africa, airborne Mn exposures at levels substantially lower than current occupational exposure thresholds in the U.S. may be associated with clinical parkinsonism.

## Data Availability

Data from research participants in this study, who authorized sharing of their research data, will be made available to investigators with appropriate expertise and research support, after publication of the primary aims of this study. All shared data will be de-identified and will be released in accordance with U.S. and South African regulations.

## Abbreviations

ACGIH: American Conference of Governmental Industrial Hygienists
CI: Confidence interval
EPA: Environmental Protection Agency
GABA: gamma aminobutyric acid
GP: Grooved pegboard.
km: Kilometers.
LOAEL: Lowest observed adverse effect level
Mn: Manganese
NATA: National-Scale Air Toxics Assessment
PD: Parkinson disease
PM: Particulate matter
SD: Standard deviation
UPDRS3: Unified Parkinson Disease Rating Scale motor subsection part 3
U.S.: United States
